# Perceived physical health outcomes of direct-acting antiviral treatment for hepatitis C: a qualitative study

**DOI:** 10.1186/s12954-021-00516-1

**Published:** 2021-07-15

**Authors:** Stelliana Goutzamanis, Danielle Horyniak, Joseph S. Doyle, Margaret Hellard, Peter Higgs, on behalf of the Treatment and Prevention Study group

**Affiliations:** 1 Disease Elimination Program, Burnet Institute, 85 Commercial Rd, Melbourne, VIC 3004 Australia; 2 School of Public Health and Preventive Medicine, Monash University, 553 St Kilda Rd, Melbourne, VIC 3004 Australia; 3 Behaviour and Health Risks Program, Burnet Institute, 85 Commercial Rd, Melbourne, VIC 3004 Australia; 4 Department of Infectious Diseases, The Alfred and Monash University, 85 Commercial Rd, Melbourne, VIC 3004 Australia; 5 Department of Public Health, La Trobe University, Plenty Rd and Kingsbury Dr, Bundoora, VIC 3086 Australia

**Keywords:** Hepatitis C, Qualitative research, Direct-acting antiviral, Treatment experience, People who inject drugs, Quality of life

## Abstract

**Background:**

Novel health promotion and treatment uptake initiatives will be necessary to ensure Australia meets 2030 hepatitis C elimination targets. Increasing treatment uptake will be assisted by a better understanding of the treatment experience and patient-perceived benefits. This study describes the perceived physical health benefits from direct-acting antiviral (DAA) hepatitis C treatment among people who inject drugs in Melbourne, Australia.

**Methods:**

Twenty participants were recruited from a community treatment trial and community health clinics. Semi-structured interviews were performed with each participant before, during and following treatment. Interviews focused on treatment experiences, attitudes and motivations. Interviews were recorded, transcribed and thematically analysed.

**Results:**

Two themes relating to the physical experience of treatment developed; intersection between physical and mental health and “maybe it’s working”. Participants reported various physical benefits, most prominently, reduced fatigue. Reductions in fatigue resulted in instant and meaningful changes in everyday life. Some participants did experience side effects, which they described as mild. Experiencing noticeable physical benefits during treatment was perceived as validation that treatment was working.

**Conclusion:**

Physical health benefits of DAA treatment may have carry-on effects on cognitive, emotional or social wellbeing and should be incorporated into how treatment is promoted to those who require it.

## Introduction

Highly efficacious direct-acting antiviral (DAA) treatment is beginning to change the epidemiology of hepatitis C virus. In Australia, DAAs are government subsidised and available to anyone; between 2016 and 2018 over 70,000 people living with hepatitis C in Australia were treated with DAAs. [1] Nevertheless, Australia will not meet World Health Organization elimination targets without increased testing and treatment. [2] People who are yet to be treated may have different motivations and priorities to those who were treated in the early roll out of DAAs, suggesting a need for novel promotion strategies to engage this group with treatment.

Importantly, the experience of undertaking DAA treatment is starkly different to that of previous interferon-based treatment. Although many clinical trials report improvements in health-related quality of life during and following DAA treatment [3], few studies have described the experience of undertaking treatment for people who inject drugs. The existing qualitative studies have focused heavily on the emotional and psychological benefits of treatment, such as reductions in uncertainty and internalised stigma [4] or increased feelings of freedom and empowerment. [5] Despite the obvious importance, the physical health benefits of treatment have received little attention. It is important that the growing body of treatment experience literature includes the full breadth of benefits available through treatment, including any physical health improvements. This will better inform health promotion and hepatitis C symptom management.

In this paper we describe physical health outcomes for people who inject drugs undertaking DAA treatment for hepatitis C, in Melbourne, Australia.

## Methods

We draw on data from a longitudinal qualitative study examining DAA treatment experiences. [6] Twenty participants were recruited from two sources: a community-based hepatitis C treatment trial and two different community health clinics. Participants had a history of injecting drug use and had either received a treatment consultation or a prescription for treatment. Semi-structured interviews were conducted with each participant before, during and following treatment, between September 2017 and July 2019 in Melbourne, Australia. Interviews were flexible and conversational and focused on the social, emotional and physical experiences of treatment, as well as attitudes towards, perceptions of and motivations for treatment. Interviews were recorded and lasted approximately 40 min. Participants were given pseudonyms which are used throughout this paper. Interviews were transcribed, then read, re-read and initial notes were made. This allowed participant specific topics to be raised in subsequent interviews. Once all interviews were complete, reflexive thematic analysis was performed. [7] This began with inductive open coding using NVivo (version 12, QSR International, Australia). All pre-treatment interview transcripts were coded first, followed by the during- and then post-treatment transcripts. Codes were combined to create themes representing patterns of treatment meaning and experience. Themes were reviewed, named and defined. A thematic map was produced to describe how themes were related. Matrices were used to describe at what time point themes developed and whether they changed over time. Reflexive journaling and discussion among co-authors occurred throughout data collection and analysis. Participants provided written informed consent prior to the first interview, and additional verbal consent for subsequent interviews. Participants were reimbursed AUD $40 for their time after each interview.

## Results

This analysis used data from 51 interviews with 19 participants who initiated DAA treatment. One participant who was recruited did not initiate treatment and so their data was not used in this analysis. Of the 19 participants included, 14 completed all three interviews. Some participants were unable to complete their final interview as they had relocated, or contact was lost. Most participants were male (*n* = 14), and reported no or a low level of fibrosis (liver scarring) at baseline (*n* = 11). However, four participants were unaware of their fibrosis status. At baseline 19 participants had injected drugs in the past month and two participants were employed. Five participants were born overseas. Participants were aged from 20 to 54 years old. Table 1 details the full list of treatment benefits and symptoms described by participants during interviews. Some of these were only reported by a single participant and others, by almost all participants. Our results focus mostly on the alleviation of fatigue, given it was the most widely reported benefit of treatment.

**Table 1:** Participants perceived hepatitis C treatment benefits and side effects (*n* = 19)

	Physical	Emotional	Cognitive
Treatment benefits	Increased appetite	Increased motivation	Clearer thinking
	Craving healthier foods	Improved mood (more positive mindset, happier, less agitated, less frustrated, calmer)	Improved focus and concentration
	Weight gain		
	Decreased sweating		Improved speech
	Decreased bloating		Improved memory
	Decreased liver pain	Sense of achievement	More alert
		Increased confidence	
	Decreased bodily pain		
	Fewer colds/illness	Decreased stigma (experienced and internalised)	
	Quicker skin wound healing		
	More energy/less fatigued	Decreased uncertainty and worry	
	Better quality sleep/less sleep required		
	Overall feeling better		
Side effects	Flatulence	None reported	None reported
	Constipation		
	Dizziness and nausea		
	Poor skin wound healing		
	Lower libido		

### Intersection between physical and mental health

Participants described experiencing a wide range of physical, emotional, and cognitive benefits during or following treatment (see Table 1 for full list). The perceived impact of treatment on physical health varied from minor to substantial, with many of the reported benefits being unique to individual participants. For some, perceived physical health improvements were specific, such as increased appetite or decreased sweating, bloating and liver pain. Such clear improvements in physical health had flow on effects for participants’ nutrition, comfort, and daily activities. However, most descriptions of treatment benefits blurred the lines between physical, cognitive, and mental domains of wellbeing. For example, some participants described a general feeling of decreased malaise following treatment.

Everything is all clear you know, and yeah my body is actually, when I had the hep C my body was so run down, because I was fighting the virus and since it’s gone my body’s gone back to functioning normally. (Hugo, post-treatment)

Overwhelmingly the intersection between physical and mental health was described in relation to fatigue. Prior to treatment participants described a multidimensional impact of fatigue where “it sort of affects everything”. For some it was difficult to explain the intensity and reach of their fatigue to others.

I get tired a lot. [My partner] can’t work out why I always go lie down for half an hour or an hour before I pick the kids up from school. It’s just all my energy feels like it’s being taken from me, and I just need to rest. (Miriam, pre-treatment)

Improved fatigue was the most frequently reported benefit from treatment. All but two participants described experiencing some degree of better quality sleep, less interrupted sleep, requiring less sleep at night or fewer naps during the day, feeling less tired throughout the day, feeling more alert or having more energy. Prior to treatment Zara described consistently waking up “feeling like I’ve had a big day already”, regardless of how well she slept. Following treatment, she described having more energy:

I’m not waking up tired. I’m a bit tired at the end of the day, at like five, six after I’ve had dinner, then I’m a bit sleepy, but before I would wake up tired. So that’s a big difference there! (Zara, post-treatment)

Having more energy was a particularly tangible and noticeable outcome of treatment. Even feeling slightly more energised had a meaningful impact on participants’ lives and relationships. Increased energy was conceptualised by what it afforded participants in their daily lives—running more errands, doing more household tasks, feeling physically stronger, exercising, finding it easier to study or work and “keeping up with the kids”. Increased energy and its manifestations in daily life were closely tied to mood, motivation and mental energy.

I guess it’s not so much apathy when I wake up…there’s not that ‘I can’t be fucked’ feeling anymore. I mean I still can’t be…but it’s less, a lot less. I get that I have to do it. It’s like this morning I wanted to go back to bed but I didn’t, I ended up getting up. (Kiran, post-treatment)

The nexus between physical and mental health was also displayed in participants’ attitudes towards treatment side effects. Participants knew it was unlikely they would experience severe side effects but expressed fears that they might be unlucky. In the pre-treatment interview some participants expressed doubts and worries about experiencing severe side effects and what that would mean beyond their physical health.

Yeah, I was worried about side effects and how it would affect me with bringing up my children, like I thought it would make me really sick where I wouldn’t be able to look after my kids and stuff. (Miriam, pre-treatment)

Despite doubts, during treatment, many participants described experiencing no major side effects associated with treatment, an experience which was met with celebration, excitement and for some surprise. A few participants did report side effects at the beginning of treatment, such as nausea and constipation (Table 1), although side-effects were largely perceived as minor and/or short lived. One participant attributed drowsiness to the treatment, which prompted concern about their ability to work.

Umm when I first started taking the tablets, there was drowsiness, like it would knock me out to sleep, but now um I got used to it, but I still don’t trust it and stuff, if I drive the forklift I’m not going to take it and drive. (Ken, during-treatment)

### “Maybe it’s working”

Noticeable changes in physical health offered participants a type of reassurance or comfort by suggesting treatment was effective and/or that previous feelings of fatigue were valid and attributable to hepatitis C.

Whilst participants praised the simplicity of treatment, they also wanted validation of treatment efficacy. Changes in physical health, either improvements or decrements, were perceived as an indication that treatment was ‘working’.

Last week, when I picked up my second bottle (pause) I seem to have a little bit more energy, which is good. Well I’m going on a lot more walks, because I just have a little bit more energy…The only way I can tell it is working is that I have more energy and I’m taking that as a positive and I’m assuming it’s the pill taking away the hep C and giving me more energy, so I hope I’m right. (Cam, during-treatment)

Conversely, experiencing no distinguishable physical health improvements or symptoms during treatment incited worry and suspicion for a few participants. Most participants regularly drew distinctions between their understandings of interferon treatments and DAA treatment. However, it seemed, for some, there may have been an underlying or subconscious belief, perhaps embedded since the interferon era that toxicity was indicative of effectiveness.

Yeah, yeah and I haven’t, I’ve had no side effects whatsoever, it’s like nothing happened, so I just want to see when I do my blood test what happens…No side effects, nothing, it’s like I’m not even taking the pill, how I was before is like how I am now. (Van, during-treatment)

Being unsure of whether treatment was working did not impact on adherence. However, for participants like Van, who were enrolled in a trial having their viral load measured at the end of treatment as well as the usual 12 weeks following treatment was reassuring.

From what I hear, I spoke to the [clinical trial nurses] yesterday and they said everything is looking good. I am so relieved, I’m so like (pause) it’s a burden. (Van, post-treatment)

Noticing improvements in fatigue also authenticated or explained previous feelings of fatigue, which alleviated some uncertainty and self-doubt. Prior to treatment many participants described being unable to pinpoint a cause of their intense fatigue, which left some participants wondering “maybe it’s just me, maybe I’m lazy”. Li didn’t reveal feeling fatigued until she noticed an improvement during treatment, which was a validating and positive experience.

Well I quietly thought to myself that sometimes I felt lethargic and I wondered if [hepatitis C] was the cause of it... I sort of feel like I have more energy, so maybe it is working. I feel happier within myself. I don’t notice being lethargic nowadays. (Li, during-treatment)

## Discussion

This report describes participant perceived physical health outcomes of DAA treatment for hepatitis C. Treatment afforded participants in our study wide-ranging benefits, which had variable impact on daily life. Some benefits were experienced by many participants and others by only a single participant, some altered the fabric of everyday life and others were perceived as minor. Participant narratives highlighted that the benefit of treatment may be variable and personal, interrelating physical, emotional, social and cognitive domains of wellbeing.

The most reported and most impactful physical health benefit was the alleviation of fatigue. Despite fatigue being highlighted as a key domain of living with hepatitis C [8], there has been little in-depth exploration of fatigue in the DAA era. Patient reported outcome data from clinical trials suggests being cured of hepatitis C is associated with an improvement in fatigue. [9] Our findings provide context to clinical trial data, describing how participants conceptualise improvements in fatigue as beyond purely a change in physical state. Improvements in fatigue manifested in various way, for example, better quality sleep, more energy throughout the day, requiring less naps etc. This altered the structure of daily life and afforded participants meaningful changes in how they physically and mentally exert themselves.

Participant narratives of minimal side effects and ease of undertaking treatment contributed to a positive perception of treatment, but also the desire to substantiate the efficacy of treatment. The presence of side effects and/or noticeable physical health improvements during treatment were taken as indication treatment was working. The perception that treatment toxicity may be a biomarker for effectiveness is not a novel notion and has been reported in the field of oncology [10] as well as hepatitis C. [11, 12] Participants recruited from the community-based treatment trial regularly commented on and appreciated the regular updates from study nurses on their treatment progress and their hepatitis C viral load. This echoes data from both interferon and DAA treatment trials, where participants described valuing clinical feedback on treatment progression, particularly being able to observe a downward trajectory in viral load, which was reassuring, motivating and satisfying. [13, 14] The simplicity of DAA treatments and knowledge around efficacy rates, does not mean everyone will experience treatment entirely free of ambiguity. There may be an important role to play, not just for clinicians but also for peers to support people undertaking treatment by offering reassurance, clear messaging around potential for side-effects or complete lack of side effects, and ensuring sustained virologic response testing (confirmation of cure) is simple and accessible for those who would like it.

The serial interviews were a unique strength of this study, allowing the experience of initiating, undertaking, and completing hepatitis C treatment to be captured at the time, instead of in retrospect. Concerns about treatment and experienced side effects may be recounted differently or downplayed when treatment has been successfully completed. Our study is limited by convenience sampling and capturing the treatment experiences of a reasonably research and healthcare engaged cohort. Additionally, approximately half the sample were enrolled in a community-based clinical trial and were receiving more frequent follow up than one would usually receive throughout treatment. This may have influenced their perceptions and knowledge about treatment. Finally, we did not explore the perceived physical health benefits of those living with cirrhosis (severe living scarring), which may differ from those with minimal fibrosis.

Our study adds to the emerging body of qualitative literature on the DAA treatment experience and provides explanation to quality of life clinical trial data. Participant narratives indicate that people experience physical benefits from treatment beyond cure and liver health improvement. This has numerous implications. Firstly, in order to provide patient centred care clinicians should seek to understand their patients perceived hepatitis C symptoms and their desired and experienced outcomes from treatment. Thus, if perceived symptoms such as fatigue persist beyond treatment, clinicians can offer appropriate avenues of treatment and management. Secondly, our findings suggest that when promoting treatment clinicians should carefully incorporate the potential for improved fatigue, without guaranteeing specific treatment outcomes, but stressing the individual and variable impact of treatment. This may be important in engaging a broader group of people in treatment, including those in early stages of infection, some who may not perceive an urgent need for treatment.

## Data Availability

Not applicable.
